# Endoanchors for the distal fixation of iliac limb in endovascular aneurysm repair

**DOI:** 10.1016/j.jvscit.2024.101700

**Published:** 2024-12-05

**Authors:** Andrea Spertino, Matteo Spezia, Francesco Squizzato, Michele Antonello

**Affiliations:** Division of Vascular and Endovascular Surgery, Department of Cardiac, Thoracic, Vascular Sciences and Public Health, University of Padua, Padua, Italy

**Keywords:** Abdominal aortic aneurysm, Endoanchor, Endoleak, EVAR, Iliac aneurysm

## Abstract

This report details the case of an 84-year-old male with an infrarenal abdominal aortic aneurysm and a dilated right common iliac artery eligible for endovascular treatment. A bifurcated stent graft (Medtronic Endurant IIs) was used to treat the aneurysm. To address the concerns of instability of the right iliac limb, four endoanchors (Heli-FX EndoAnchor, Medtronic) were placed at the distal landing zone to provide additional fixation. This case shows good result in the improvement of stability of the iliac limb with potentially enhanced durability.

Endovascular aneurysm repair (EVAR) has become the gold standard for treating abdominal aortic aneurysms (AAAs). Despite significant advancements in EVAR techniques, challenges persist, especially regarding the secure proximal fixation in hostile neck and tourtuos dilated iliac arteries.^1^ In cases of short or angulated necks, traditional stent graft deployment may be suboptimal, increasing the risk of migration and endoleaks. Endoanchors have emerged as a novel approach to address these challenges by providing additional fixation points for the endograft.^2^

Type Ib endoleaks have been reported to occur in 8% of cases when certain anatomical characteristics are present, such as a large common iliac artery diameter, a short landing zone, and significant iliac tortuosity, necessitating adjunctive procedures to mitigate this complication.^3^

This case report presents a patient with complex aortic anatomy who underwent EVAR with the peculiar use of endoanchors both for proximal neck and distal iliac limb fixation.

## Case report

An 84-year-old male was admitted with an infrarenal AAA and a right common iliac artery aneurysm. His medical history included atrial fibrillation, hypertension, pulmonary emphysema, stroke related to left internal carotid occlusion, appendicectomy for peritonitis, and right carotid endarterectomy. Although the patient had multiple comorbidities, he was considered for surgery because of the optimal functional capacities.

**Fig 1:**
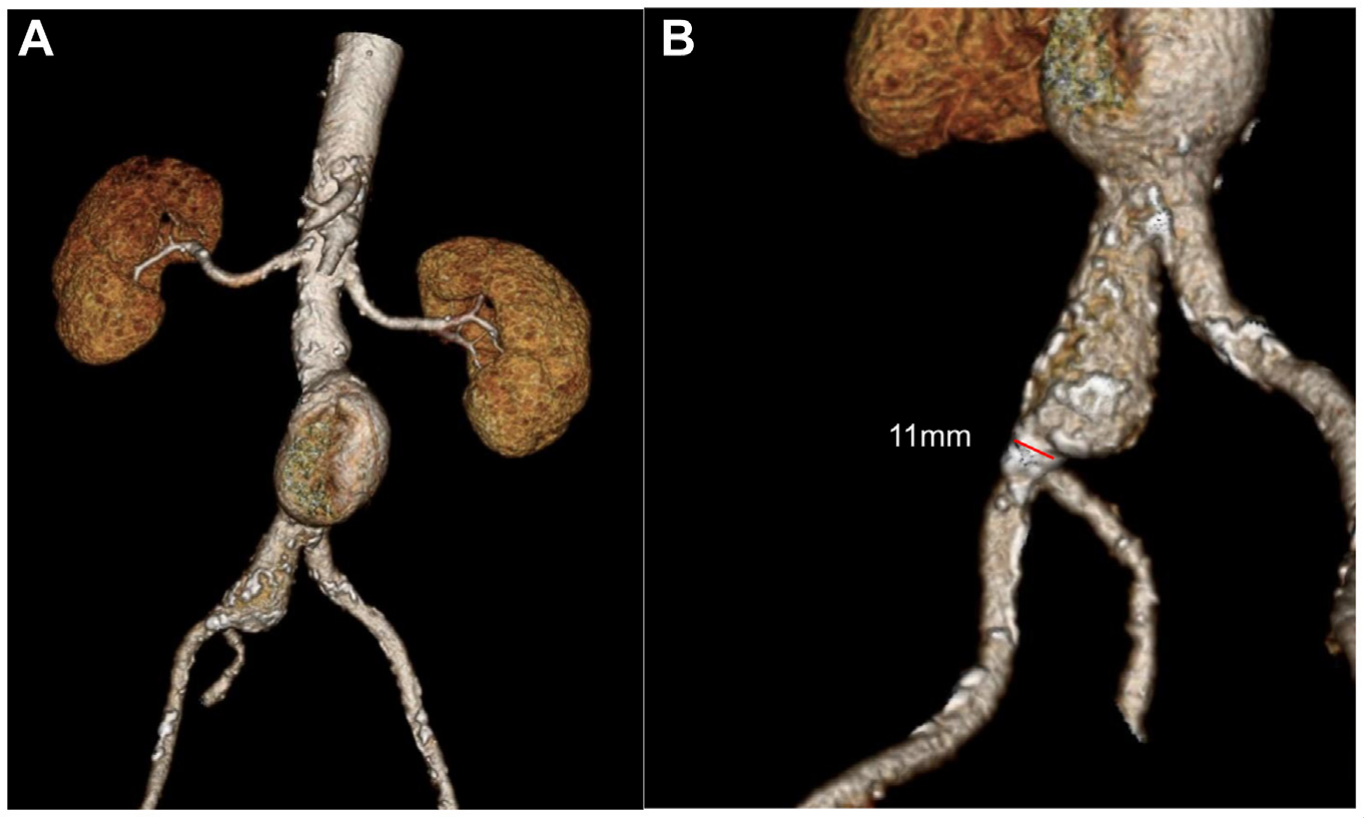
Preoperative computed tomography angiography (CTA) three-dimensional reconstruction shows the aortic saccular aneurysm **(A)**. The right common iliac artery presented some anatomical features that could expose to patient to higher risk of type IB endoleak: its maximum diameter was 24 mm with a conical shape. It also presented a narrowing to 11 mm just before the hypogastric artery origin **(B)**.

Preoperative computed tomography angiography (CTA) showed an infrarenal saccular aortic aneurysm with maximum aortic transverse diameter of 57 mm, and a short aortic proximal neck (10 mm), a right common iliac artery dilatation with a transverse diameter of 24 mm with no adequate distal landing zone, a stenosis at the origin of the right hypogastric artery, and the occlusion of the left hypogastric artery (Supplementary Video 1, online only; Fig 1).

Given the high surgical risk related to multiple comorbidities and the challenging anatomy, an endovascular approach was elected as the preferred treatment.

The patient provided informed consent for the use of Heli-FX endoanchors for the fixation of the distal gate of the right iliac limb, and was made aware that the use of this system was not on the instructions for use for the product.

Under general anesthesia, ultrasound guided retrograde common femoral artery puncture was performed bilaterally. Two Proglide devices (Abbott Vascular Inc) were placed on angles in pre-close fashion on each side. A bifurcated stent graft 23 × 14 × 103 mm (Endurant IIs, Medtronic) was deployed, completed by the left iliac limb 16 × 13 × 124 mm and the right iliac limb 16 × 24 × 94 mm.

**Fig 2:**
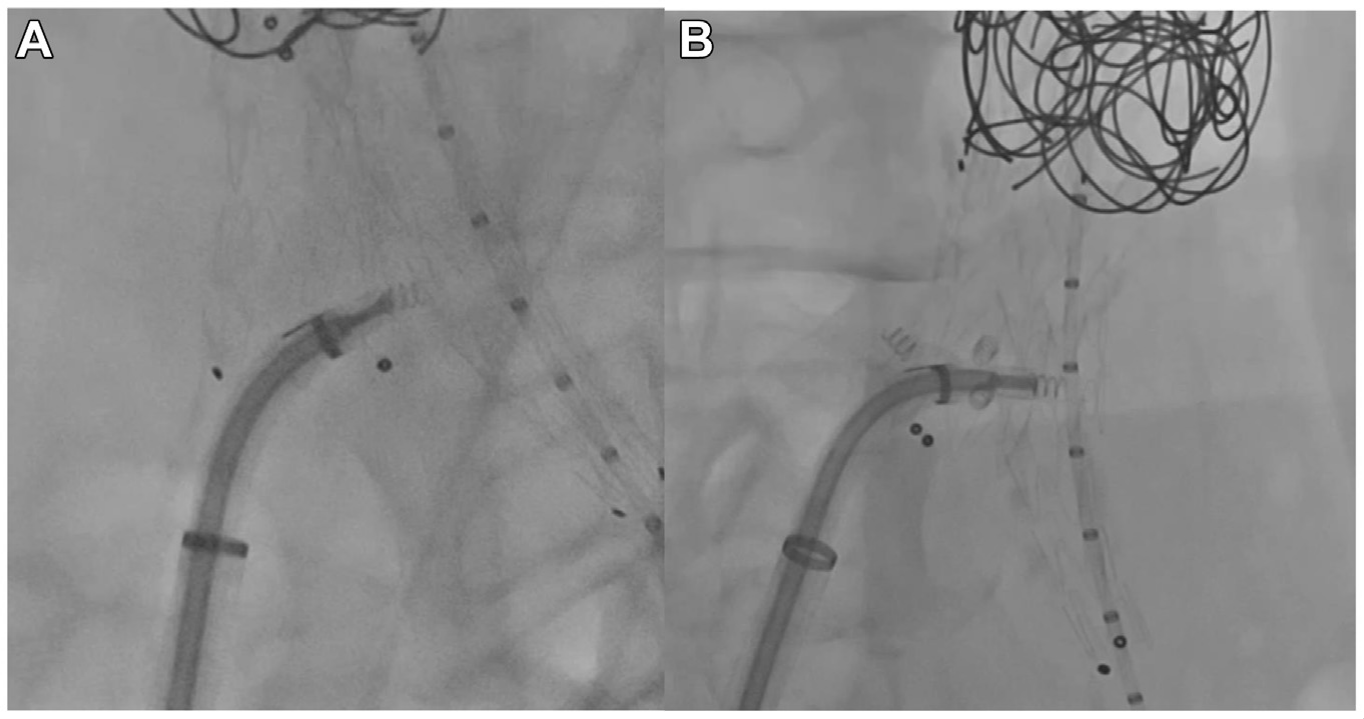
Heli-FX endoanchor system with the Heli-FX steerable guide sheath was used to secure the distal segment of the right iliac limb. It is crucial to assess the optimal orientation of the delivery system to obtain equal distance between endoanchors. The four endoanchors have been delivered, placing the steerable guidesheat orthogonally to angiographic view: two of them have been released in 45° left anterior oblique view **(A)**, and the others in 45° right anterior oblique view **(B)**.

**Fig 3:**
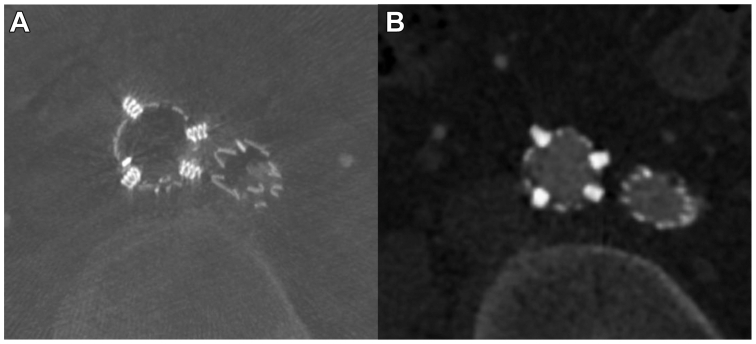
**A,** Cone beam computed tomography (CT) with CO_2_ performed at the end of the procedure shows the correct placement of the endoanchors. **(B)** Six months follow-up angio-CT scan confirms the correct placement with no evidence of migration and no signs of endoleak.

Due to the presence of two hypertrophic lumbar arteries and the lack of sac thrombus, embolization of the aneurism sac was performed using 10 hydrocoils (Azur; Terumo Corp). Six endoanchors (Heli-FX EndoAnchor, Medtronic) using the Heli-FX Guide and Heli-FX endoanchor system were deployed at the EVAR proximal sealing zone, whereas four were deployed at the right iliac limb distal sealing zone to provide additional fixation (Fig 2). A final cone-beam CT with CO_2_ was performed, showing the correct placement of the endoprosthesis and the endoanchors (Fig 3, *A*).

Postoperatively, the patient had an uncomplicated course. A follow-up CTA performed 3 months later demonstrated optimal results, with complete exclusion of the AAA with no signs of endoleak. The endoanchors were well-positioned (Fig 3, *B*), and there were no signs of iliac limb migration (Supplementary Video 2, online only). The patient will undergo standard ultrasound follow-up after the first CTA.

The patient has provided informed consent for the publication of this case report, including the use of its medical information and data.

## Discussion

This case highlights the feasibility of using endoanchors for distal iliac limb fixation in EVAR. In patients with complex aortic anatomy, traditional stent graft deployment may be associated with increased risks of migration and endoleaks. Endoanchors offer a promising approach to enhance the stability of the iliac limb, potentially improving long-term outcomes.^3^

The use of endoanchors in EVAR is a relatively new concept, and the available evidence is limited. However, initial studies have shown promising results demonstrating that endoanchors can improve graft fixation in challenging anatomical conditions, such as short or angulated aortic necks, with emerging evidence suggesting that preventive use of endoanchors may reduce the incidence of type Ia endoleaks.^4^^,^^5^ Qamhawi et al analyzed the results of the use of endoanchors for the treatment of type Ia endoleak in 60 patients. Evidence of their use with this indication is still inconsistent, whereas alternative strategies such as proximal endograft extension still have better outcomes.^6^

Although there are data about the use of Heli-Fx endoanchors in preventing the incidence of type Ia endoleaks, the use of endoanchors in the prevention of type Ib endoleaks of the common iliac artery limbs is not yet demonstrated, and this is not included in the instructions for use of the device.^7^^,^^8^

Sariciliar et al published a series of four patients where Heli-FX endoanchors were used to treat type Ib common iliac artery endoleak of EVAR grafts. With a technical success of 100% and good results at follow-up, Heli-FX endoanchors were demonstrated to be a viable option to treat type Ib endoleaks rather than alternatives such as an iliac bifurcation extensions or coil-and-cover approach.^9^

Alternatives were considered for this specific case. Current guidelines^10^ do not recommend any specific surgical approach for iliac artery aneurysm, leaving the choice based on individual patient and lesion characteristics. The coil-and-cover approach was not feasible due to contralateral hypogastric artery occlusion, with a high risk for the patient of developing pelvic ischemia. Iliac branch devices were neither adequate due to the small distal neck (11 mm) of the aneurysm on the common iliac artery before the bifurcation. The bell bottom technique was considered the more appropriate; however, there is a lack of long-term durability data, and current evidence has not addressed the concerns. To improve the long-term results, we decided to enhance distal fixation with endoanchors.

To our knowledge, to date, our case is the first one with the use of endoanchors in the prevention of type 1b endoleak. In our experience, a guide sheath with a deflected tip reach of 22 mm was used with a 24-mm distal gate of the iliac limb, and no difficulties were encountered delivering the endoanchors in the iliac limb, even if they are not designed for this purpose. The potential pitfalls in delivering endoanchors in the iliac limb could be related to mismatch between the delivery system angulation tool and the iliac limb diameter.

Despite these promising results in the treatment and prevention of type Ib endoleaks, it is important to note that long-term outcomes of endoanchors use are still unknown.^9^^,^^11^ Further studies are needed to evaluate the durability of the technique and identify potential complications. Although this case demonstrates a successful outcome, larger studies are needed to evaluate the efficacy and safety of endoanchors in a broader patient population. Long-term follow-up is essential to assess the durability of the technique and identify potential complications.

## Conclusion

The use of endoanchors for distal iliac limb fixation in EVAR offers a potential solution for managing challenging aortic and iliac anatomy. This case report demonstrates the feasibility and potential benefits of this technique. However, further experience is needed to establish the optimal indications and long-term outcomes.

## Funding

None.

## Disclosures

None.

## Appendix (online only)

Supplementary Video 1 (online only) Preoperative computed tomography angiography (CTA) in axial view.

Supplementary Video 2 (online only) Six-month follow-up computed tomography angiography (CTA) in axial view showing the good result of the intervention with no signs of endoleaks. There is no evidence of right iliac limb and endoanchors displacement.
